# β-catenin mediates monocrotaline-induced pulmonary hypertension via glycolysis in rats

**DOI:** 10.1186/s12872-024-04000-z

**Published:** 2024-07-23

**Authors:** Hui Meng, Yan Deng, Juan Liao, Dan-dan Wu, Li-xiang Li, Xing Chen, Wei‑Fang Lan

**Affiliations:** 1 Department of Ultrasound, The First Affiliated Hospital of Guangxi Medical University, Nanning, People’s Republic of China; 2 Department of Echocardiography of Cardiovascular Disease Institute, First Affiliated Hospital of Guangxi Medical University, 6 Shuang Yong Road, Nanning, 530021 People’s Republic of China

**Keywords:** β-catenin, Glycolysis, Monocrotaline, Macrophages, NLRP3 inflammasome, Pulmonary hypertension

## Abstract

**Background:**

Metabolic abnormalities and immune inflammation are deeply involved in pulmonary vascular remodelling and the development of pulmonary hypertension (PH). However, the regulatory mechanisms of glycolysis in macrophages are still elusive. Cumulative evidence indicates that β-catenin plays a crucial role in metabolic reprogramming. This study aimed to investigate the effect of β-catenin on macrophage glycolysis in PH.

**Methods:**

LPS-induced BMDMs were generated via in vitro experiments. A monocrotaline (MCT)-induced PH rat model was established, and the β-catenin inhibitor XAV939 was administered in vivo. The role of β-catenin in glycolysis was analysed. The degree of pulmonary vascular remodelling was measured.

**Results:**

β-catenin was significantly increased in both in vitro and in vivo models. In LPS-induced BMDMs, β-catenin increased the levels of hexokinase 2 (HK2), phosphofructokinase (PFK), M2-pyruvate kinase (PKM2), lactate dehydrogenase (LDH), and lactate (LA) and the expression of inflammatory cytokines and promoted PASMC proliferation and migration in vitro. XAV939 decreased the level of glycolysis and downregulated the expression of inflammatory cytokines in vivo. MCT promoted pulmonary arterial structural remodelling and right ventricular hypertrophy, and XAV939 alleviated these changes.

**Conclusions:**

Our findings suggest that β-catenin is involved in the development of PH by promoting glycolysis and the inflammatory response in macrophages. Inhibition of β-catenin could improve the progression of PH.

**Supplementary Information:**

The online version contains supplementary material available at 10.1186/s12872-024-04000-z.

## Introduction

PH is a severe cardiopulmonary disease that arises from a combination of factors [1]. PH is characterized by a progressive increase in pulmonary vascular resistance, leading to irreversible pulmonary circulation disorder, right ventricular overload, and ultimately, right heart ventricular failure and premature mortality [2]. The prevalence of PH is estimated to be approximately 1% of the global population, with a higher incidence of up to 10% in individuals older than 65 [3]. Despite the significant impact of PH, current clinical treatment options are limited and often inadequate. Targeted therapies, such as prostaglandins, phosphodiesterase-5 inhibitors, endothelin receptor antagonists, and soluble guanylate cyclase stimulators, primarily focus on modulating the endothelin, nitric oxide, and prostacyclin pathways [4, 5]. Although these drugs can improve haemodynamics and relieve patients' symptoms, they are not effective in delaying the pulmonary vascular remodelling process or fundamentally curbing PH progression [5]. Therefore, further studies on novel therapeutics might provide an experimental basis and a theoretical basis for the clinical treatment of PH.

Abnormal metabolism, especially aerobic glycolysis, has been identified as an important mechanism of PH [6]. It promotes cell proliferation, migration, and an antiapoptotic tumour-like malignant phenotype, leading to pulmonary vascular remodelling, right ventricular hypertrophy, and the acceleration of PH [7]. Furthermore, glycolysis impacts the ability of macrophages to differentiate and polarize, which may cause the activation of the NLRP3 inflammasome and amplify the inflammatory response [8, 9]. Our previous studies demonstrated the improvement of PH progression upon NLRP3 inhibition [10–12]. Therefore, blocking the reprogramming of glucose metabolism in macrophages can effectively improve the development of PH. Many enzymes participate in glycolysis, but the rate of glycolysis in macrophages is influenced mainly by the content of glucose and key enzymes, including HK2, PFK, PKM2 and LDH [8]. Therefore, the state of cell glycolysis can be defined by detecting the above indicators.

The available evidence indicates that the Wnt/β-catenin pathway is involved in regulating glycolysis, inflammation, and immune regulation [13–15]. Moreover, in the classical Wnt cascade, β-catenin is the key effector protein for the transduction of signals to the nucleus, and it triggers the transcription of Wnt-specific genes that control and determine the fate of many cells [16]. β-catenin promotes the abnormal proliferation, invasion and apoptosis of inflammatory cells and tumour cells by regulating their metabolism, leading to the development of many diseases [17, 18]. Here, we hypothesize that β-catenin could participate in PH by upregulating glycolysis.

To explore the role of β-catenin in PH, we administered MCT to establish in vivo models of PH [19]. By causing endothelial dysfunction, MCT effectively mimics the inflammation-related PH observed in clinical settings [20]. In the present study, we demonstrated that β-catenin is a regulator of glycolysis. Increased glycolysis in macrophages promoted by β-catenin contributes to the inflammatory response in pulmonary hypertension, and inhibition of β-catenin may become a new therapeutic target for PH.

## Materials and methods

### Experimental animals and ethics statement

Pathogen-free inbred male Sprague–Dawley rats (220–250 g) were obtained from the Experimental Laboratory Animal Center of Guangxi Medical University (Nanning, China). The rats were housed at standard room temperature (21–22 °C) and humidity (60–65%). All animals were provided adequate access to food and water. All the experimental protocols and procedures were performed in compliance with the National Institutes of Health Guidelines on the Use of Laboratory Animals (NIH Publication No. 85–23, revised 1996) and were approved by the Institutional Animal Care and Use Committee (Application number: 201901010) of Guangxi Medical University (Nanning, China).

### Rat model of MCT-induced PH and experimental groups

The PH rat model was established by a single intraperitoneal injection of MCT (60 mg/kg). MCT was sufficiently dissolved in 1 N HCl, 0.5 N NaOH was added to neutralize the solution to pH 7.4, and the solution was then diluted with distilled water. A total of 24 male rats were randomly assigned to four experimental groups (*n* = 6/group): (1) the saline + vehicle group (sham group), (2) the saline + XAV939 group (sham + XAV939 group), (3) the MCT + vehicle group (MCT group), and (4) the MCT + XAV939 group (MCT + XAV939 group). The dose of XAV939 (5 mg/kg/d) used in the study was based on a previous report [21]. After MCT administration, XAV939 in phosphate-buffered saline (PBS) was randomly injected intraperitoneally for 21 days.

### Echocardiography

Twenty-eight days after MCT administration, echocardiography and haemodynamic evaluations were performed on all rats. The rats were anaesthetized with Nembutal. Echocardiography was performed using a Resona 7 system with an L20-5U transducer (Mindray M7, Shenzhen Mindray Biomedical Electronics Co. Ltd., China). The tricuspid annular plane systolic excursion (TAPSE), RV end diastolic dimension (RVEDD) and right ventricular ejection fraction (RVEF) were calculated by the following formulas: RVEF (%) = (RVEDV—RVESV)/RVEDV × 100%. The TAPSE was measured by M-mode echocardiography. The measurements were recorded from 10 consecutive beats and were used to normalize the beat-to-beat variations.

### Haemodynamic measurements and tissue processing

The RVSP was measured by thoracotomy compression. Briefly, rats were anaesthetized and placed on a thermostatic heating pad at 37 °C, intubated and connected to a ventilator (tidal volume 5.0 ml, respiratory rate 80 breaths/min). The surgeon punctured the right heart immediately after opening the chest and then connected it to a pressure sensor. The data were collected by the cardiac pressure volume system ADV500 (Transonic, Beijing, China) through a computer. The animals were euthanized after the completion of RVSP data collection. Hearts from the rats were rapidly excised and weighed. The dry weights of the right ventricular (RV) free wall and the left ventricle plus septum (LV + S) were measured, and then the RV/LV + S ratio was calculated. Portions of the right ventricle and lung tissue from all experimental groups were fixed in 4% paraformaldehyde before being paraffin-embedded for histopathological examination. Some lungs were excised for ELISA, glycolysis-related index measurements and Western blotting (stored at -80 °C).

### Histological analysis

Paraffin sections were made with lung tissue. First, the sections were stained with haematoxylin–eosin (HE). The medial wall thickness was determined in pulmonary arteries and arterioles with outer diameters of 50–100 μm. Occlusion was calculated by the following formula: (total area-lumen area)/total area × 100%. Then, the segments were incubated with α-smooth muscle actin (α-SMA, 1:150, BIOSS, Beijing, China) antibodies overnight at 4 °C, washed in PBS and incubated with HRP-labelled goat anti-mouse/rabbit IgG polymer for 30 min. After incubation with horseradish for 10 min and DAB staining for 3–5 min, the samples were completely rinsed with tap water, and haematoxylin and anti-blue were added, and the samples were dehydrated, clarified and sealed. Images were examined using an Olympus BX51 microscope. The thickness of the PA wall or smooth muscle layer was measured in cross-sectional images of the lung, and the remodelling of the pulmonary artery (PA) was quantified by morphometric analysis of distal small PAs (50–100 μm). Each lung slice had 10 random selections of eligible vessels. ImageJ software (NIH, Bethesda, MD) was used to measure the luminal area of arterioles.

### Primary rat PASMC and pulmonary macrophage (PM) isolation

Rat PASMCs obtained from the medial smooth muscle layer of pulmonary arteries were isolated as described previously [22]. Briefly, rat pulmonary artery samples were cut longitudinally with ophthalmic scissors, and the inner and outer membranes were removed by gentle scraping 2 to 3 times with a blade and rinsing with PBS. The PA was cut into 1 mm^3^ pieces and placed in the bottom of a T-25 culture flask. The flask was incubated at 37 °C with 5% CO2 for 3–4 h, and then 3 mL of Dulbecco's modified eagle medium (DMEM) containing 20% foetal bovine serum(FBS) was added to the flask. After 5–7 days of culture, a small number of cells grew from the edge of the tissue block. Once the cells reached approximately 70% confluence, PASMCs with good growth were harvested from the second through sixth passages and used for subsequent experiments. Anti-α-SMA immunohistochemical staining was used to identify PASMCs.

Primary rat PMs were collected by bronchoalveolar lavage as described previously [23]. Briefly, the lungs of rats were harvested for multiple lavages with PBS, and the rat alveolar lavage fluid was recovered. After centrifugation (1000 rpm for 5 min), the red blood cells were removed, and the cells were lysed for 5 min. Subsequently, the PMs were isolated and cultured in Iscove's modified Dulbecco's medium (IMDM) supplemented with 20% FBS. Anti-CD68 immunohistochemical staining was used to identify PMs 3 days later.

### Bone marrow-derived macrophage (BMDM) isolation and culture

As previously described, rats weighing between 150–180 g were anaesthetized via intraperitoneal injection of 3% Nembutal. The entire femur was rapidly excised under aseptic conditions, and the adherent periosteum and muscle tissues were meticulously removed before transferring the bone to a sterile ultraclean workstation. The femur was then cleansed with PBS supplemented with 1% penicillin–streptomycin, after which both metaphyseal ends were amputated. The bone marrow cavity was subsequently flushed alternately with IMDM from each end, and the resulting rinse fluid was collected. After filtration through a 70 µm cell sieve, the suspension was centrifuged at 1000 rpm for 10 min. The supernatant was discarded, and an appropriate volume of red blood cell lysis buffer was added to the pellet. The cells were resuspended and allowed to stand for 5–10 min before undergoing a second centrifugation. Again, the supernatant was discarded.

For routine culture, the cells were resuspended in complete IMDM containing 10% FBS, 100 U/mL penicillin, 100 µg/mL streptomycin, and 20 ng/mL macrophage colony-stimulating factor (M-CSF). The culture medium was refreshed every three days. The macrophages were induced to mature on days 6 to 7, after which they were utilized for subsequent experimental procedures.

### Cell treatments

On the 6th day of BMDM culture, interventions were conducted. The β-catenin agonist lithium chloride (LiCl) and the selective glycolytic activator d-fructose 1,6-bisphosphate trisodium salt octahydrate (FBP) served as negative controls for the in vitro therapeutic efficacy of XAV939. The BMDMs were divided into five groups: the control group (diluent), LPS group, LPS + XAV939 (10 mM, Selleck, USA) group, LPS + LiCl (10 mM, Selleck, USA) group, and LPS + XAV939 + FBP (4 mM, Alladin, Shanghai, China) group. After the cells were pretreated with XAV939 and LiCl for 48 h, the cells were stimulated with LPS (100 ng/ml) for 6 h. In the LPS+XAV939+FBP group, FBP was added 24h before lsp treatment. After treatment, the BMDMs were collected for Western blot analysis, and the supernatants were collected for ELISA. To study the effect of inflammatory cytokines secreted by BMDMs on PASMCs, the supernatants from these BMDM cultures were used to treat PASMCs for 24 h. Subsequently, CCK-8, scratch, EdU, and Transwell assays were performed on PASMCs to assess the effects of the supernatants on PASMC proliferation, migration, and invasion.

Second, to verify that glycolysis in macrophages promotes the activation of the NLRP3 inflammasome, we used 2-deoxy-D-glucose (2-DG, a competitive inhibitor of glucose metabolism) and FBP. The BMDMs were divided into a control group (diluent), an LPS group, an LPS + 2-DG (4 mM, MCE) group, an LPS + FBP (4 mM, Alladin, Shanghai, China) group. Following a 6-h administration of LPS (100 ng/ml), 2-DG and FBP were administered.

### Cell Counting Kit-8 (CCK-8) assay

A CCK-8 assay kit (Dojindo Laboratory, Kumamoto, Japan) was used to determine PASMC viability according to the manufacturer’s instructions. Briefly, cells were plated at a density of 2.0 × 10^3^ cells/well in 96-well plates. After the corresponding treatment, cell proliferation was assessed with a CCK-8 kit according to the manufacturer’s instructions. The number of cells was determined relative to that of the control cells in triplicate experiments.

### PFK and LDH activities and content of glucose and LA determination

PFK and LDH activities and glucose and LA levels were determined by commercially available colorimetric assay kits (Solarbio, Beijing, China) according to the manufacturer's instructions.

### ELISA

Cell culture supernatants and rat lung tissue homogenates were analysed after centrifugation. The levels of TNF-α, IL-6, IL-1β and IL-18 were measured using commercially available ELISA kits (Jiang Lai creatures, Shanghai, China) according to the manufacturer’s instructions.

### Western blot analysis

Tissue and cellular proteins were extracted using RIPA lysis buffer (Solarbio, Beijing, China) supplemented with protease and phosphatase inhibitors (Beyotime, Shanghai, China). The concentrations of the extracted proteins were measured using a BCA Protein Assay Kit (Beyotime, Shanghai, China). Equal amounts of protein were separated by 10% SDS–polyacrylamide gel electrophoresis and transferred onto nitrocellulose membranes. The membranes were probed with antibodies against β-catenin (1:1000, Proteintech, Wuhan, China), HK2, PKM2 (1:4000, Proteintech, Wuhan, China), ASC, pro-caspase-1, caspase-1, NLRP3 (1:1000; all Cell Signaling Technology, USA), β-actin (1:2500, Proteintech, Wuhan, China). After blocking with 5% (w/v) dry milk for 1 h, antibodies were added and incubated overnight at 4 °C. After the membranes were washed, they were incubated with horseradish peroxidase-conjugated secondary antibodies (1:5000; ZSGB-Bio, Beijing, China) for 2 h. PVDF was immersed in sensitized ECL and then exposed to X-rays via a gel imaging system (UVP, Upland, CA) to develop the protein bands. ImageJ software (National Institutes of Health, Bethesda, MD) was used to quantify the density of the bands, which were normalized to β-actin levels.

### 5-Ethynyl-2'-deoxyuridine (EdU) cell proliferation assay

PASMCs were cocultured with BMDM supernatants from each group for 24 h. PASMC proliferation was detected by an EdU incorporation assay. PASMCs were stained with the BeyoClick™ EdU Apollo488 In Vitro Imaging Kit (Beyotime, Shanghai, China) according to the manufacturer’s instructions. Nuclei were stained with Hoechst.

### Cell migration

Cell migration was determined by a scratch wound assay and transwell assay. For the wound assay, PASMCs were cultured in six-well plates in starvation medium and wounded with a sterile pipette tip to generate a cell-free gap of 1 mm width, and the wound location in the culture dish was marked as previously described. The cells were photographed to record the wound width at 0 h. Afterwards, the cells were treated as previously described. Twenty-four hours later, photographs were taken again at the marked wound location for migration measurement. For the transwell assay, PASMCs (1 × 10^5^) were seeded into the upper chamber at 8 μm/well (1 × 10^5^ cells/well) in serum-free medium. Then, BMDM supernatants from each group were inoculated into the lower chamber. The cells were cultured at 37 °C and 5% CO2 for 24 h. The migrated cells were fixed with 4% paraformaldehyde at room temperature for 30 min and stained with crystal violet dye (Biyuntian, Shanghai, China) for 20 min. Images were captured for quantification.

### Statistical analysis

GraphPad 9.0 software was used for all the statistical analyses and bar chart production. The data are expressed as the mean ± standard deviation (SD). Comparisons between two groups (with a normal distribution) were performed by unpaired Student’s t tests. One-way analysis of variance (ANOVA), followed by the Newman–Keuls multiple comparison test for normally distributed data, was used for comparisons among multiple groups. Statistical significance was characterized as ns *P* ≥ 0.05, *P < 0.05, **P < 0.01, ***P < 0.001, and ****P < 0.0001 (two-tailed).

## Results

### Increased expression of β-catenin in PMs from rats with MCT-induced PH

**Fig. 1:**
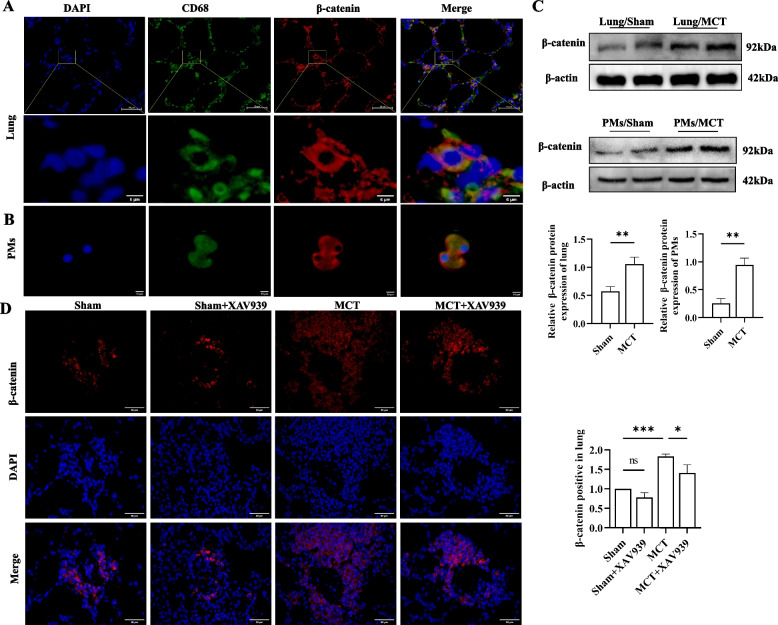
β-catenin is localized in PMs and is expressed at high levels in MCT-induced PH. **A**-**B** Double immunofluorescence staining showing the localization of β-catenin expressed on macrophages in MCT-induced lungs. **A** Paraffin sections of lung tissue and **B** primary macrophages cultured by alveolar lavage. The top panels show low-magnification images of DAPI (blue), β-catenin (red), and CD68 (green) staining. Scale bar = 50 μm. The bottom panels present high-magnification insets of the yellow boxes in the top panels. **C** Western blots and quantification analysis showing the protein levels of β-catenin in lung tissue and primary pulmonary macrophages. The blots were cropped and hybridized with the corresponding antibodies. Quantitative Western blot results were normalized to those of β-actin. **D** Immunofluorescence staining and quantification analysis showing the expression of β-catenin (red). The results of quantitative immunofluorescence staining were normalized to those of DAPI. Sham, Sham group; Sham + XAV939, Sham + XAV939 group; MCT, MCT group; MCT + XAV939, MCT + XAV939 group. The data are presented as the means ± SDs; ns *P* ≥ 0.05, *P < 0.05, **P < 0.01, ***P < 0.001, ****P < 0.0001; *n* = 3/group

To examine the distribution and expression of β-catenin in PH, double immunofluorescence staining was used to determine the localization of β-catenin in MCT-induced PH rats' lungs, and the results showed that β-catenin was mainly distributed in PMs (Fig. 1A). Importantly, we isolated and cultured PMs, repeated double fluorescence staining and obtained the same results (Fig. 1B). Western blotting showed that β-catenin protein levels were significantly increased in both lung tissue and PMs in the MCT-induced model group compared to those in the sham group (Fig. 1C). Additionally, the immunofluorescence results showed that XAV939 significantly downregulated the expression of β-catenin in the lungs of MCT-induced PH rats (Fig. 1D). These results demonstrate that β-catenin might play a role in the progression of PH in PMs.

### β-catenin enhances glycolysis and the activation of the NLRP3 inflammasome in LPS-induced BMDMs

**Fig. 2:**
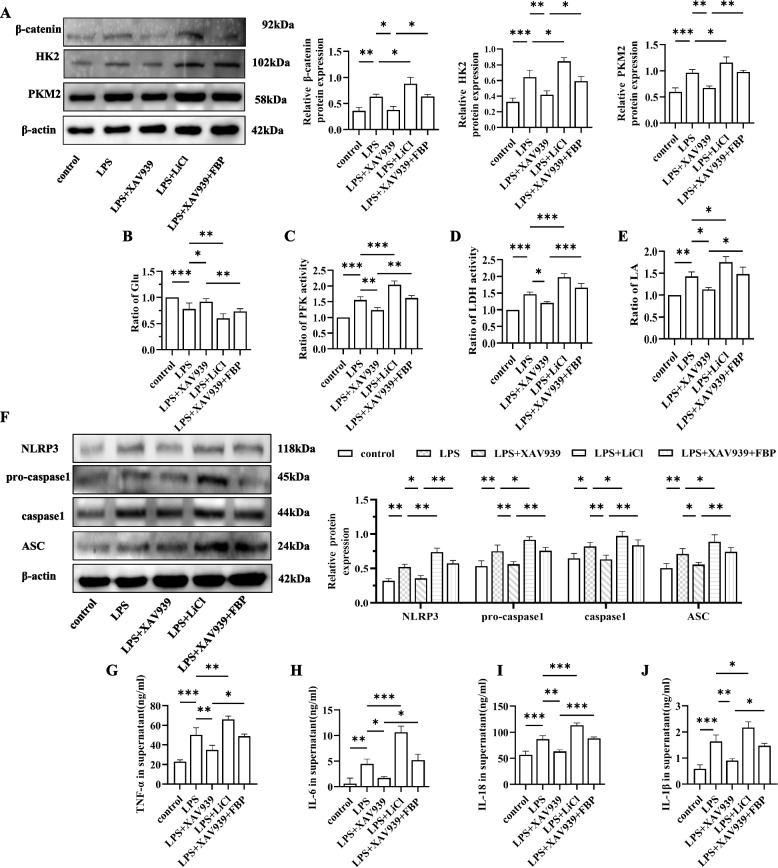
β-catenin enhances glycolysis and NLRP3 inflammasome activation in vitro. **A** Western blot showing the expression of β-catenin,HK2 and PKM2 in BMDMs. The blots were cropped and hybridized with the corresponding antibodies.Quantitative Western blot results were normalized to those of β-actin. **B**-**D** Glucose,PFK and LDH measurements in BMDMs. **E** Lactate determination in the cell supernatant. **F** Representative Western blot showing the expression of NLRP3 inflammasome markers in BMDMs from the different groups. β-Actin was used as the internal reference. **G**-**J** TNF-α, IL-6, IL-1β and IL-18 levels in the culture supernatants of BMDMs were measured by ELISA. The following comparisons were made: control vs. LPS, LPS vs. LPS + XAV939, LPS vs. LPS + LiCl, and LPS + XAV939 vs. LPS + XAV939 + FBP. The data are presented as the means ± SDs; ns *P* ≥ 0.05, *P < 0.05, **P < 0.01, ***P < 0.001, ****P < 0.0001; *n* = 3/group

To determine the importance of β-catenin in the regulation of macrophage glycolysis, we conducted in vitro cell assays using BMDMs. First, Western blotting revealed that the protein level of β-catenin was significantly greater in the LPS group than in the control group. Importantly, the content of β-catenin in the XAV939 group was reduced, while it appeared to be the highest in the LPS + LiCl group (Fig. 2A). A colorimetric assay revealed decreased glucose content in the BMDMs of the LPS group, while the levels of key enzymes such as PFK, LDH and the glycolytic product LA were significantly increased compared to those in the control group (Fig. 2B-E). Importantly, the protein expression levels of the other two key enzymes, HK2 and PKM2, were also greater than those in the control group (Fig. 2A). These findings indicated that LPS stimulation could increase glycolysis in BMDMs. After the application of XAV939, the increasing trends of HK2, PFK, PKM2, LDH and LA were reversed in the LPS group. At the same time, after treatment with FBP, a glycolytic agonist, the inhibition of glycolysis by XAV939 was reduced, as indicated by the upregulation of key glycolytic enzymes and the increase in LA (Fig. 2B-E). Thus, we concluded that XAV939 directly inhibits glycolysis in BMDMs. Moreover, the level of glycolysis in BMDMs was significantly enhanced after treatment with LiCl, a β-catenin agonist, which also indicated that the positive effect of β-catenin on glycolysis was clear.

Previous reports have suggested the involvement of β-catenin in NLRP3 inflammasome activation [24], and inhibition of β-catenin reduces inflammatory responses. To further investigate the effect of β-catenin on NLRP3 activation in macrophages, we measured the protein levels of the NLRP3 inflammasome in BMDMs. Compared with those in the control group, the levels of NLRP3, ASC, pro-caspase-1, and caspase-1 were increased in the LPS group. However, compared with those in the LPS group, the levels of these indicators were lower in the LPS + XAV939 group and greater in the LPS + LiCl group. Moreover, the levels of key glycolytic enzymes and NLRP3 inflammasome-related proteins were greater in the LPS + XAV939 + FBP group than in the LPS + XAV939 group. Consistently, the cytokine levels (TNF-α, IL-6, IL-1β and IL-18) in the supernatants of macrophages from each group demonstrated a similar trend (Fig. 2F-J). Based on these findings, we concluded that β-catenin contributes to the activation of the NLRP3 inflammasome in BMDMs.

### Enhanced glycolysis promotes the activation of the NLRP3 inflammasome in LPS-induced BMDMs

**Fig. 3:**
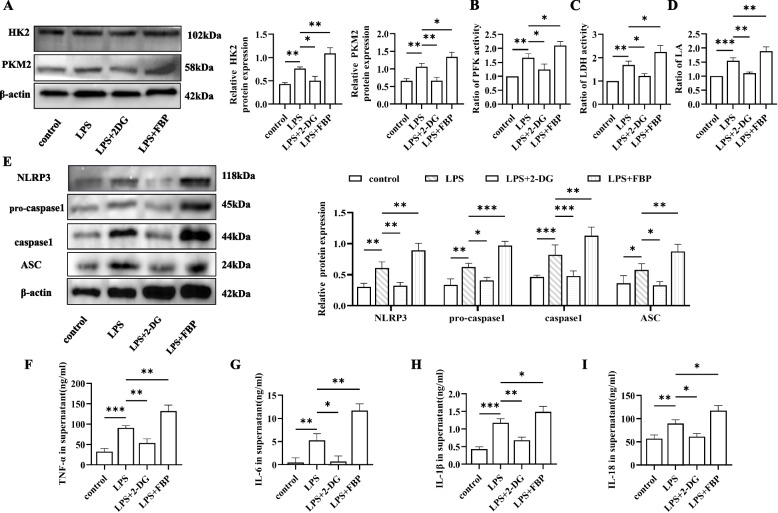
Enhanced glycolysis promotes the activation of the NLRP3 inflammasome in vitro. **A** The expression of HK2 and PKM2 in BMDMs. β-Actin was used as the internal reference. **B**-**C** The levels of the key glycolytic enzymes PFK and LDH were detected in vitro. **D** Lactate determination in the cell supernatant. **E** Representative Western blot showing the expression of NLRP3 inflammasome markers in BMDMs from the different groups. The blots were cropped and hybridized with the corresponding antibodies. β-Actin was used as the internal reference. **F**-**I** TNF-α, IL-6, IL-1β and IL-18 levels in the culture supernatants of BMDMs were measured by ELISA. The following comparisons were made: control vs. LPS, LPS vs. LPS + 2-DG, and LPS vs. LPS + FBP. The data are presented as the means ± SDs; ns *P* ≥ 0.05, *P < 0.05, **P < 0.01, ***P < 0.001, ****P < 0.0001; *n* = 3/group

Next, we verified that glycolysis in LPS-exposed BMDMs promoted the activation of the NLRP3 inflammasome in vitro. The cells were grouped based on their corresponding stimulation, and lactate levels and key glycolytic enzyme levels were measured to confirm successful intervention in all groups (Fig. 3A-D). Western blotting and ELISA analyses were then performed. Compared with those in the control group, the levels of NLRP3 inflammasome-related proteins and key glycolytic enzymes in the LPS group were increased. However, after administration of the glycolytic inhibitor 2-DG, the protein expression of the NLRP3 inflammasome was inhibited, whereas the levels were upregulated in the FBP-treated group compared to those in the LPS group (Fig. 3E). Furthermore, the levels of the inflammatory factors detected above in the cell culture supernatants by ELISA followed the same trend (Fig. 3F-I). These findings indicate that macrophage glycolysis is involved in the activation of the NLRP3 inflammasome.

### Inhibition of β-catenin suppresses PASMC proliferation and migration

**Fig. 4:**
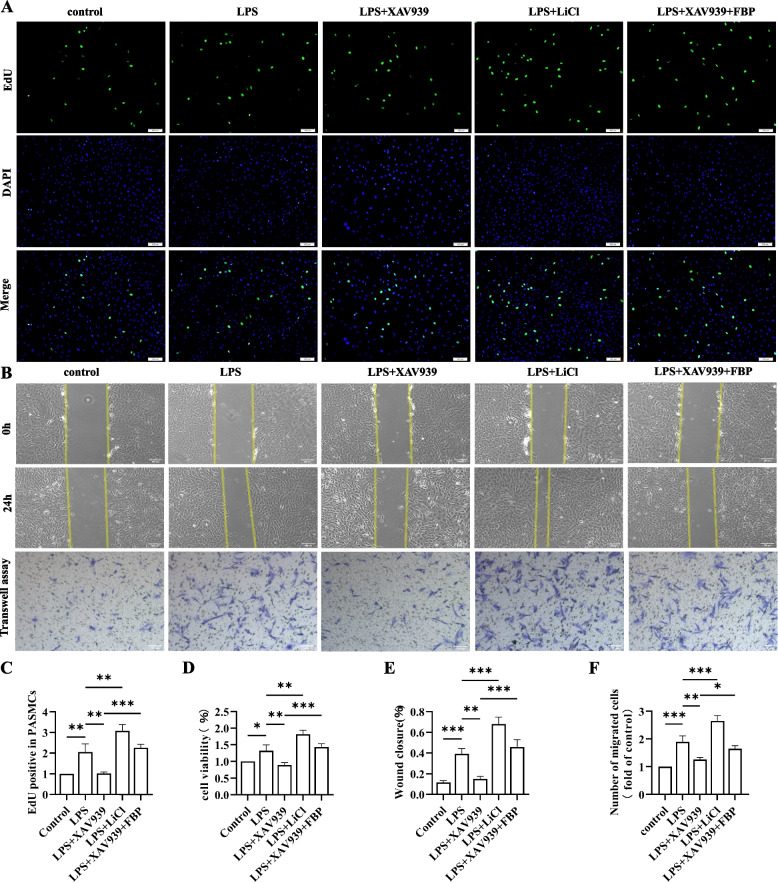
Inhibition of β-catenin suppresses PASMC proliferation and migration in vitro. **A** PASMC proliferation was assessed using the EdU assay. Scale bar = 50 μm. **B** Cell migration were assessed by wound healing and Transwell assays. Scale bar = 200 μm. **C**-**F** Bars represent the proliferation of PASMCs as assessed by EdU, CCK-8, wound healing and Transwell assays. The following comparisons were made: control vs. LPS, LPS vs. LPS + XAV939, LPS vs. LPS + LiCl, and LPS + XAV939 vs. LPS + XAV939 + FBP. The data are presented as the means ± SDs; ns *P* ≥ 0.05, *P < 0.05, **P < 0.01, ***P < 0.001, ****P < 0.0001; *n* = 3/group

Abnormal proliferation of PASMCs is an important mechanism for PH. To investigate the effect of β-catenin on the proliferation and migration of PASMCs, we established a coculture system of BMDM supernatants and PASMCs. Subsequently, the PASMCs were subjected to EdU, CCK-8, fluorescence detection scratch, and Transwell migration assays. EdU and CCK-8 assays showed that PASMC viability was greatest in the LPS + LiCl group, while PASMC vitality decreased in the LPS group after XAV939 treatment (Fig. 4A-C). Moreover, the results from the 24-h scratch assays revealed that PASMCs treated with supernatants from the LPS + LiCl group exhibited the highest rate of wound healing, followed by those from the LPS group and LPS + XAV939 + FBP group. The same trend was observed in the Transwell migration assay, as the number of migrating cells in the LPS + XAV939 group decreased compared to that in the LPS group (*P* < 0.001) (Fig. 4D-F). Taken together, these results suggest that inhibition of β-catenin effectively attenuated the proliferation and migration of PASMCs.

### XAV939 alleviates haemodynamics, pulmonary vasculature and right ventricular remodelling in MCT-induced PH

**Fig. 5:**
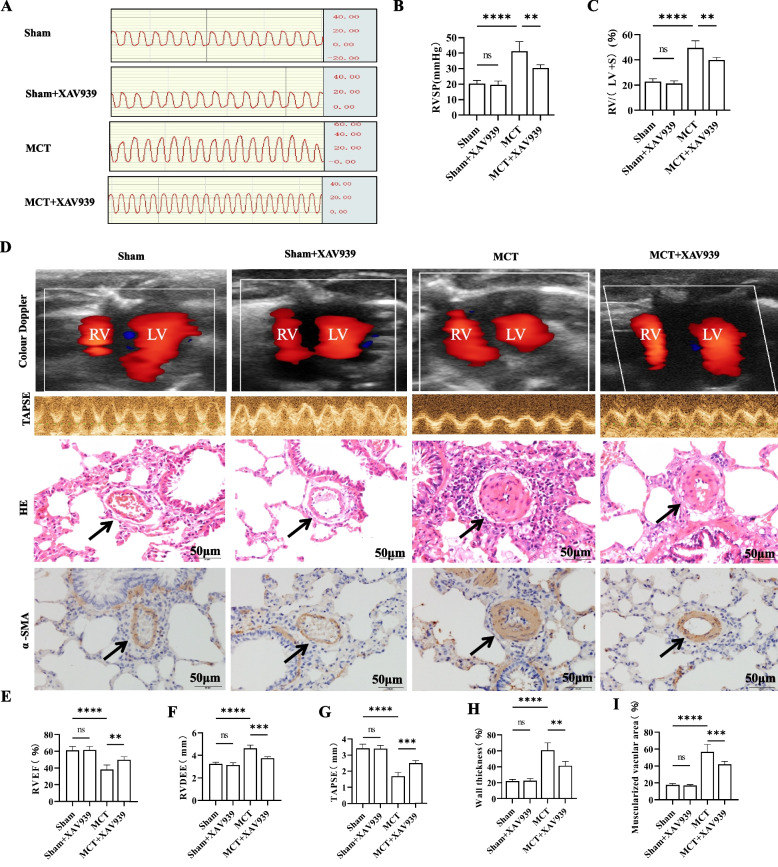
XAV939 attenuated the development of MCT-induced PH in rats. **A** Right ventricular pressure measurement in rats. **B** Bar graph comparing RVSP levels in the different groups of rats. **C** XAV939 pretreatment reduced the RV/LV + S ratio in MCT-treated rats. **D** Representative images of colour Doppler, TAPSE, HE and immunostaining of α-SMA in the RV in the Sham, Sham + XAV939, MCT, and MCT + XAV939 groups. Scale bar = 50 μm. **E**–**G** Quantification of RVEF, RVEDD, and TAPSE via echocardiography. **H**-**I** Graph showing the percentage of the median arteriole thickness by HE staining and the percentage of muscularization by α-SMA immunostaining. The data are presented as the means ± SDs; ns *P* ≥ 0.05, *P < 0.05, **P < 0.01, ***P < 0.001, ****P < 0.0001; *n* = 6/group

Haemodynamic measurements were taken 28 days after MCT administration, revealing a significant increase in RVSP in MCT-exposed rats compared to those in sham rats. However, treatment with XAV939 effectively reduced this phenomenon (Fig. 5A-B). Furthermore, the structure and function of the RV were examined. The increase in RV/(LV + S) in MCT-treated rats was attenuated by XAV939 (Fig. 5C). Transthoracic cardiac ultrasound revealed that rats exposed to MCT demonstrated a noteworthy increase in RVEDD, along with reductions in TAPSE and RVEF, in comparison to those in the control group. However, the administration of XAV939 effectively reversed these effects (Fig. 5D-G). Additionally, histological examination revealed that XAV939 reduced pulmonary vascular wall thickening caused by MCT. HE staining showed the percentage of the median arteriole thickness was lower in MCT+XAV939 group than in the MCT group(Figure 5D, H). Immunohistochemistry for α-SMA, a smooth muscle cell marker, demonstrated a decrease in the increase in the muscularization rate of pulmonary blood vessels induced by MCT after treatment with XAV939 (Fig. 5D, I). These findings indicate that XAV939 mitigates alterations in RV structure and enhances RV function in a rat model of PH.

### XAV939 reduces glycolysis and suppresses the NLRP3 inflammasome and proinflammatory cytokines in MCT-exposed rats

**Fig. 6:**
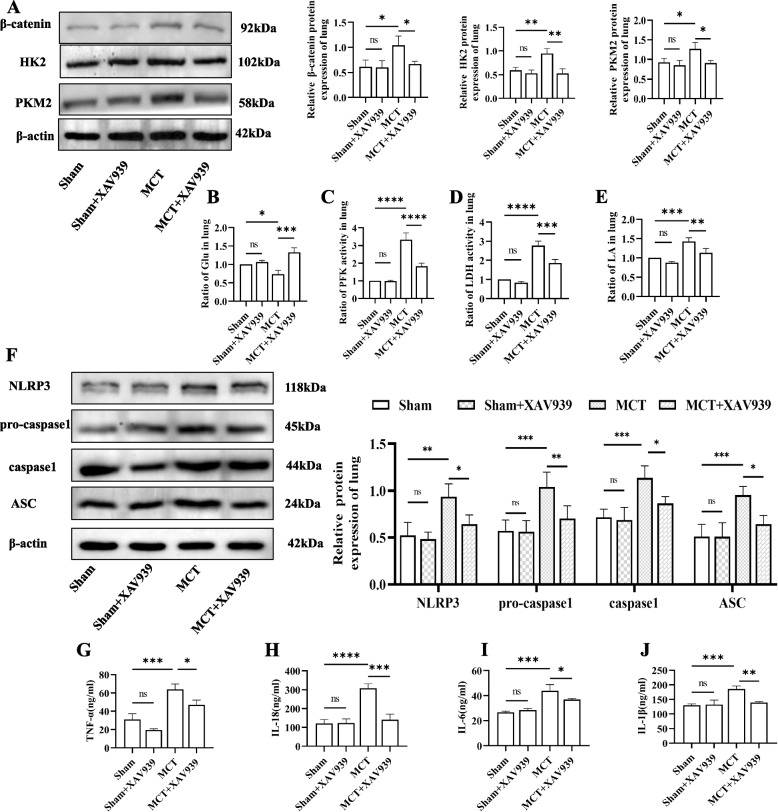
XAV939 suppressed glycolysis and NLRP3 inflammasome activation in vivo. **A** Western blot showing the expression of β-catenin,HK2,PKM2 in vivo. Quantitative Western blot results were normalized to those of β-actin. **B** The glucose content in lung tissue from the four groups of rats. **C**-**E** The contents of PFK, LDH and LA in vivo. **F** Representative Western blot showing the expression of NLRP3 inflammasome markers in lung samples from the different groups. The blots were cropped and hybridized with the corresponding antibodies. β-Actin was used as the internal reference. **G**-**J** The levels of the proinflammatory cytokines TNF-α, IL-6, IL-1β and IL-18 in the lung tissue supernatants of the rats. The following comparisons were made: Sham vs. Sham + XAV939, Sham vs. MCT, and MCT vs. MCT + XAV939. The data are presented as the means ± SDs; ns *P* ≥ 0.05, *P < 0.05, **P < 0.01, ***P < 0.001, ****P < 0.0001; *n* = 3/group

Western blotting revealed successful inhibition of β-catenin in the MCT + XAV939 group compared with the MCT group (Fig. 6A). The expression levels of the glycolytic enzymes HK2, PFK, PKM2, and LDH were increased in the MCT group, indicating a reprogramming of glucose metabolism in these rats (Fig. 6A-C). Colorimetric assay analysis revealed a notable difference in lactate levels content between the MCT groups and the sham groups (Fig. 6D). Specifically, the MCT group exhibited significantly elevated lactate levels compared to those in the sham group.Interestingly, treatment with XAV939 in our inhibition-of-function study attenuated the aforementioned changes compared to those in the MCT-induced PH model rats. XAV939 significantly decreased the levels of the NLRP3 inflammasome markers NLRP3, pro-caspase-1, caspase-1 and ASC after inhibiting glycolysis (Fig. 6E). Additionally, the ELISA results showed a reduction in the levels of proinflammatory cytokines, such as TNF-α, IL-6, IL-1β and IL-18, in the lung tissue supernatants of rats in the MCT + XAV939 group compared to those in the MCT group (Fig. 6F-I). These findings indicate that XAV939 can directly block glycolysis and decrease the expression of the NLRP3 inflammasome and proinflammatory cytokines in MCT-treated rats.

## Discussion

The role of β-catenin in the pathogenesis of various diseases, including PH, has been extensively studied. It has been reported that β-catenin promotes the abnormal proliferation, invasion and apoptosis of inflammatory cells, leading to the development of many diseases [17, 18]. However, its specific contribution to PH remains unclear. In this study, we demonstrated that (1) the upregulation of β-catenin in a rat model of PH induced by MCT and the β-catenin inhibitor XAV939 has a protective effect against PH. Furthermore, (2) β-catenin mediates glycolysis and promotes the activation of the NLRP3 inflammasome in macrophages. (3) β-catenin increased the secretion of the inflammatory cytokines IL-18, IL-1β and TNF-α and promoted PASMC proliferation and migration. Our findings suggest that β-catenin plays a crucial role in the development of PH by promoting glycolysis and increasing inflammatory responses and PASMC proliferation and migration.

PH is a group of chronic progressive diseases characterized by inflammation and immune dysregulation. The increased inflammation and immune cell infiltration in the lungs of PH patients strongly support the involvement of inflammation in the pathogenesis of this condition. Additionally, abnormal metabolism, particularly aerobic glycolysis or the Warburg effect, has been implicated in the development of PH. Previous studies have shown that alterations in glycolytic enzymes and their metabolites contribute to the regulation of innate immune function and the inflammatory response [6, 25, 26]. Some researchers even describe the relationship between glycolysis and inflammation as a “close criminal partnership.” Specifically, key enzymes of glycolysis are directly involved in the initiation, assembly, maturation, and secretion of the NLRP3 inflammasome through various pathways [27]. In our study, we found that glycolysis positively influenced the activation of the NLRP3 inflammasome. The glycolytic agonist FBP enhanced the activation of the NLRP3 inflammasome and increased the secretion of inflammatory factors, while the glycolytic inhibitor 2-DG decreased the secretion of inflammatory factors. Therefore, blocking glycolysis can effectively inhibit the inflammatory response.

The Wnt/β-catenin pathway is crucial for maintaining tissue homeostasis, and its dysregulation has been associated with various severe lung diseases, such as chronic obstructive pulmonary disease (COPD), lung inflammation, idiopathic pulmonary fibrosis (IPF), hyperoxic injury, bronchopulmonary dysplasia (BPD), silicosis, and lung cancer [28]. Previous studies have reported that activation of the Wnt/β-catenin pathway leads to increased glycolysis [29–31], which means that β-catenin inhibition could attenuate glycolysis levels. As expected, we observed an attenuated effect of XAV939 on glycolysis in our study. XAV939 downregulates the expression of key glycolytic enzymes, including HK2, PKM2, and LDH. Importantly, we found that XAV939 alleviated inflammation in vivo and in vitro, which is consistent with the findings of previous studies indicating that sustained activation of β-catenin drives inflammatory and fibrotic changes in the lungs [32].

However, the involvement of β-catenin in lung inflammation is complex and may be related to the status of PMs. Activation of the Wnt/β-catenin signalling pathway can inhibit PM self-renewal and promote inflammation [33]. Several studies have shown that the interaction between β-catenin and hypoxia-inducible factor 1 alpha (HIF-1α), a key regulator of glycolysis metabolism, promotes glycolysis-dependent inflammation in PMs while inhibiting mitochondrial metabolism and PM proliferation [33, 34]. It may also participate in inflammation through processes such as apoptosis and autophagy [14, 35]. Furthermore, the crosstalk between Wnt/β-catenin signalling and the NLRP3 inflammasome is an important aspect to consider. In our study, we demonstrated that treatment with XAV939 reduced glycolysis and the expression of NLRP3 inflammasome-associated proteins and inhibited the secretion of inflammatory factors. Moreover, agitating β-catenin in macrophages increased the proliferation and migration of PASMCs. These findings suggest that β-catenin is responsible for inflammation and fibrosis in the lungs. By mediating critical processes such as glycolysis, inflammatory responses, fibrosis and vascular remodelling, β-catenin could contribute to the progression of PH.

However, it is important to acknowledge the limitations of our study. First, we utilized only the MCT-induced PH model in our experiments, and it would be valuable to validate our results in other PH models, such as hypoxic rats, hypoxic mice, and hypoxia + SU5416-treated rats. Second, considering the physiological differences between mice and humans, it is crucial to further validate our findings in clinical studies. Third, our study focused on the high expression of β-catenin in the lungs, and it would be beneficial to investigate whether this phenomenon occurs in other organs, such as the kidney and liver. However, further investigations are needed to understand the function of β-catenin in PH and its impact on pulmonary artery remodelling.

## Conclusion

In conclusion, the findings of this study elucidated the crucial role of β-catenin in regulating the inflammatory response by mediating glycolysis. β-catenin inhibition in macrophages may represent a target for novel PH treatments.

### Supplementary Information

Supplementary Material 1.

Supplementary Material 2.

## Data Availability

The datasets supporting the findings of this article are included within the paper and its supporting file.

## Abbreviations

PH: Pulmonary hypertension
NLRP3: NOD-like receptor family pyrin domain containing 3
PASMC: Pulmonary artery smooth muscle cells
FBS: Foetal bovine serum
ELISA: Enzyme-linked immunosorbent assay
TNF-α: Tumor necrosis factor alpha
IL-1β: Interleukin-1β
HK2: Hexokinase 2
PKM2: M2-pyruvate kinase
LA: Lactate
PFK: Phosphofructokinase
LDH: Lactate dehydrogenase
