# Supplementary material for: β-catenin mediates monocrotaline-induced pulmonary hypertension via glycolysis in rats
Source: BMC Cardiovasc Disord. 2024 Jul 23;24:381. doi: 10.1186/s12872-024-04000-z (PMC11264393; doi:10.1186/s12872-024-04000-z)

Figure 1C
β-catenin

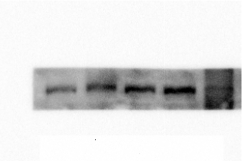

Figure 1C
β-actin

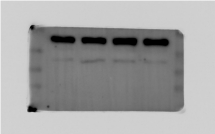

Figure 1D
β-catenin

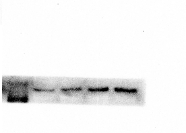

Figure 1D
β-actin

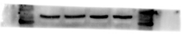

Figure 2A
β-catenin

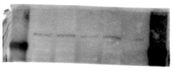

Figure 2A
β-actin

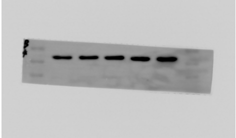

Figure 2A
HK2

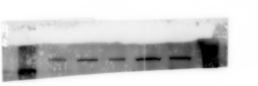

Figure 2A
PKM2

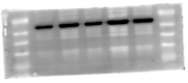

Figure 2F
NLRP3

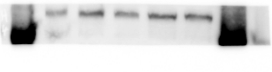

Figure 2F
pro-caspase1

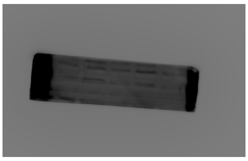

Figure 2F
caspase1

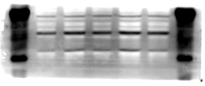

Figure 2F
ASC

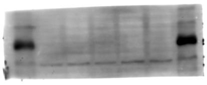

Figure 2F
β-actin

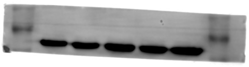

Figure 3A
HK2

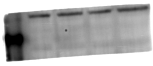

Figure 3A
PKM2

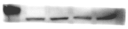

Figure 3A
β-actin

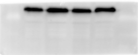

Figure 3E
NLRP3

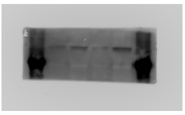

Figure 3E
pro-caspase1

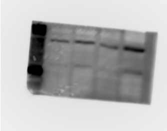

Figure 3E
caspase1

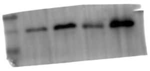

Figure 3E
ASC

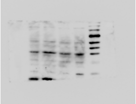

Figure 3E β-actin

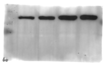

Figure 6A
β-catenin

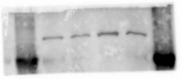

Figure 6A
HK2

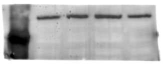

Figure 6A
PKM2

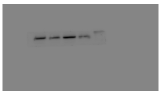

Figure 6A
β-actin

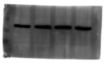

Figure 6F
NLRP3

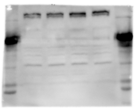

Figure 6F
Pro-caspase1

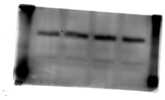

Figure 6F
caspase1

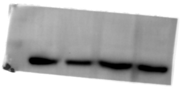

Figure 6F
ASC

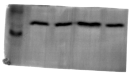

Figure 6F
β-actin

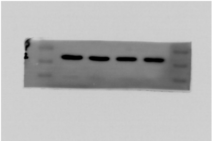

Supplement: Supplementary file 1 — Supplementary Material 1. [file 12872_2024_4000_MOESM1_ESM.pdf]
